# Determine the Complete Configuration of Single‐Walled Carbon Nanotubes by One Photograph of Transmission Electron Microscopy

**DOI:** 10.1002/advs.202206403

**Published:** 2023-03-25

**Authors:** Yue Yu, Yifan Zhao, Shouheng Li, Chao Zhao, Weiming Liu, Shanshan Wang, Feng Ding, Jin Zhang

**Affiliations:** ^1^ Center for Nanochemistry Beijing Science and Engineering Center for Nanocarbons Beijing National Laboratory for Molecular Sciences College of Chemistry and Molecular Engineering Peking University Beijing 100871 P. R. China; ^2^ School of Materials Science and Engineering, Ulsan National Institute of Science and Technology Ulsan 44919 South Korea; ^3^ Science and Technology on Advanced Ceramic Fibers and Composites Laboratory College of Aerospace Science and Engineering National University of Defense Technology Changsha 410000 P. R. China; ^4^ Faculty of Materials Science and Engineering/Institute of Technology for Carbon Neutrality Shenzhen Institute of Advanced Technology, Chinese Academy of Sciences 1068 Xueyuan Blvd Shenzhen 518055 P. R. China

**Keywords:** carbon nanotubes, complete structure, controlled growth, handedness, transmission electron microscopy

## Abstract

Developing a convenient method to determine the complete structure of single‐walled carbon nanotubes (SWNTs) is important to achieve the fully controlled growth of this nanomaterial. However, approaches that can identify handedness at the atomic level with simple equipment, operation, and data analysis are still lacking. Here, the SWNTs/graphene (Gr) vertical heterostructures are artificially constructed with aligned interfaces to realize the lattice interpretation of SWNT upper and lower walls separately by only one transmission electron microscopy image, thus transforming the 3D handedness information to projected 2D space. Gr displays prominent out‐of‐plane deformation at the interface, promoting the energetic advantage for the aligned interface construction. The interfacial alignment between the SWNT and Gr shows no obvious dependence on either the helical angle or diameter of SWNTs. The half‐wrapping of SWNTs by deformed Gr also triggers diversified alterations in electronic structures based on theoretical calculations. 27 specimens with SWNTs prepared by two disparate methods are examined, implying equal handedness distribution in the randomly aligned SWNTs grown on quartz and potential handedness enrichment in horizontal SWNT arrays grown on a‐sapphire. This work provides a simple strategy for chiral discrimination and lays a characterization foundation for handedness‐selective growth of nanomaterials.

## Graphical abstract

A convenient method to resolve the complete structure of single‐walled carbon nanotubes (SWNTs), including chiral indices (*n,m*) and handedness, using only one transmission electron microscopy image has been developed. It makes use of SWNT/graphene van der Waals heterostructures with aligned interfaces to differentiate the SWNT upper and lower surfaces, thus transforming the 3D handedness information to projected 2D space.

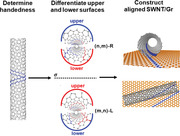

## 1 Introduction

Chirality is a significant concept in chemistry, which refers to a geometric feature of an object that is not superimposable on its mirror image. It provides materials with unique performances, including optical activities,^[^ ^1^, ^2^, ^3^, ^4^ ^]^ enantioselective separation,^[^ ^5^, ^6^, ^7^ ^]^ asymmetric catalysis,^[^ ^8^ ^]^ piezoelectricity,^[^ ^9^ ^]^ and special interactions with biological systems.^[^ ^10^ ^]^ Single‐walled carbon nanotubes (SWNTs) are promising nanomaterials for next‐generation integrated circuits beyond silicon and energy‐efficient nanotransistors.^[^ ^11^ ^]^ They can be considered as rolling up a graphene (Gr) sheet along a certain direction to form a nanometer‐scale tubular structure. Therefore, the majority of SWNTs (except armchair and zigzag nanotubes) adopt chiral structures, which include chiral indices (*n, m*) and handedness. In recent years, significant progress has been achieved in the direct growth of SWNTs with controlled density, diameter, direction, conductivity, and even chiral indices.^[^ ^12^, ^13^, ^14^, ^15^, ^16^, ^17^ ^]^ However, handedness control remains unresolved, which becomes the biggest obstacle to realizing the ultimate goal of single‐structure SWNT growth. One important reason lies in the lack of convenient and reliable characterization approaches to determine SWNT handedness, which hinders the timely feedback on the product chiral configuration of every experiment, thus making rational optimization of the growth recipe infeasible. Therefore, developing a simple, swift, and unambiguous method, which is accessible to researchers even in the field of synthesis, to identify the complete configuration of SWNTs, especially handedness, is urgently desired.

Several methods have been developed to determine the chiral structure of SWNTs. Scanning probe microscopy (SPM), including scanning tunnelling microscopy (STM) and atomic force microscopy (AFM), is capable to probe the benzene ring arrangement at the upper surface of SWNTs, thus unlocking the tube helical angle.^[^ ^18^ ^]^ However, the complete structure of SWNTs is difficult to be determined solely by SPM, but commonly requires the associated application of other characterization techniques, such as resonant Raman spectroscopy.^[^ ^19^, ^20^ ^]^ It also requires locating the same SWNT, which is only nanometer in width, when the sample is transferred from one instrument to the other. Liu et al. newly developed a Rayleigh scattering circular dichroism spectroscopic technique that can identify both chiral indices and handedness of SWNT at a single‐tube level.^[^ ^21^ ^]^ This method relies on a specifically designed setup, and SWNTs should be suspended across a 30 µm wide slit, making special requests on sample preparation. Qin et al. proposed an electron diffraction method to determine the SWNT handedness by analyzing the shift of the principle layer lines.^[^ ^22^ ^]^ However, the existence of torsion in the nanotube is the premise of this method. Transmission electron microscopy (TEM) imaging allows unambiguous identification of material's local handedness at the atomic level. Several cutting‐edge approaches were developed. Green et al. reported an exit plane wave restoration method that enables to identify both chiral indices and handedness of SWNTs.^[^ ^23^ ^]^ Suenaga et al. determined the handedness of double‐walled carbon nanotubes by capturing a tilt‐series of high‐resolution images.^[^ ^24^ ^]^ These methods restore the 3D configuration of SWNTs, where chiral information is stored, by taking either a focal series or a tilt‐series of images, which puts forward higher requirements on the experimental operation and data analysis that may not be accessible to most synthesis groups daily. Moreover, Meyer et al. recently reported the atomic‐scale topography of SWNT/Gr heterostructure,^[^ ^25^ ^]^ indicating the aligned carbon‐carbon interfaces as a preferable structure, which gave inspiration to our work.

In this work, we experimentally develop an approach to resolve the complete structure, including chiral indices (*n,m*) and handedness, of SWNTs using only one photograph taken by a simple transmission electron microscope (TEM). This method provides atomic‐scale information in real space and needs no focal series or a tilt‐series of TEM images, which is straightforward for structural interpretation and simple for experimental implementation. The method relies on the construction of SWNT/Gr van der Waals heterostructures with aligned interfaces. It enables to break the structural information degeneracy along the electron beam direction and transforms the handedness information of SWNT, which is a 3D structural feature, to a projected 2D space so that sample tilting does not necessitate. The interfacial topography of SWNT/Gr heterostructures is interpreted by a combination of TEM imaging and density functional theory (DFT) calculations, revealing the contact mode, atomic registries, and the electronic band structures at the heterojunction region. The interfacial alignment between the SWNT and Gr shows no obvious dependence on the helical angle and diameter of the carbon nanotube. This method has been successfully applied to analyze SWNTs with different morphologies by disparate preparation methods, implying equal handedness distribution in the randomly aligned SWNTs grown on quartz and potential handedness preference in horizontal SWNT arrays grown on a‐sapphire.

## 2 Results and Discussion

### 2.1 Complete Structural Determination of SWNTs

**Figure 1:**
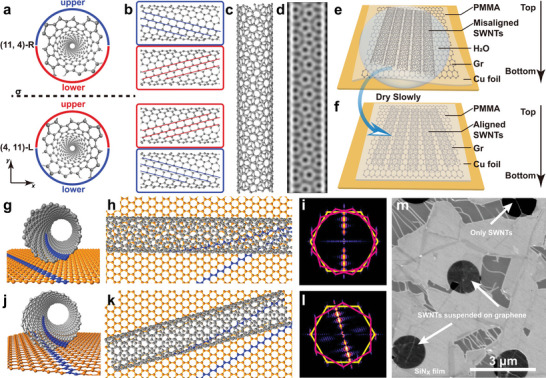
SWNT handedness determination by the construction of the SWNT/Gr heterostructure with the aligned interface. a) Perspective views along the nanotube axes showing the atomic models of a pair of SWNT enantiomers, (11,4)‐R and (4,11)‐L, respectively. The mirror plane between them is represented by a black dashed line with a label of *σ*. The electron beam injection direction is labeled as y, along which the TEM image is captured. b) Projective views of the upper and lower walls of the (11,4)‐R and (4,11)‐L SWNTs, respectively. Representative zigzag directions are highlighted by the blue and red lines. c) Projective view displaying the atomic model of either a (11,4)‐R or a (4,11)‐L SWNT. Their top views look the same. d) Simulated TEM image based on the model in (a). e,f) Schematic illustration depicting the SWNT/Gr heterostructure construction process. g‐i) 3D perspective view (g), 2D projective view (h), and the corresponding simulated diffraction pattern (i) for the atomic model of the SWNT/Gr van der Waals heterostructure with an arbitrary interface configuration. The representative zigzag orientations in both Gr and the SWNT lower wall are highlighted in blue in (g) and (h), indicating the generation of an unaligned interface. j‐l) 3D perspective view (j), 2D projective view (k), and the corresponding simulated diffraction pattern (l) for the atomic model of the SWNT/Gr van der Waals heterostructure with an aligned interface. m) SEM image displaying the SWNTs/Gr van der Waals heterostructures transferred on a holey SiN_x_ TEM grid.

The assignment of chiral indices (*n,m*) by TEM has been well documented, which commonly includes the analysis of the SWNT reflexes in reciprocal space combined with the diameter measurement and the image simulation.^[^ ^26^, ^27^ ^]^ However, the difficulty lies in the identification of handedness using only one TEM image without sample tilting or focal image series. **Figure** **1**a‐e explains the reason. Figure 1a displays the atomic models of the (11,4)‐R and (4,11)‐L SWNTs, which are mirror‐symmetry‐related enantiomorphic structures with right‐ and left‐handedness, respectively (Figure S1, Supporting Information). If regarding the SWNT as a splicing of an upper and a lower curved Gr nanoribbon, respectively, marked as the red and blue arcs in Figure 1a, the (11,4)‐R SWNT atomic model can be divided into two parts with the projective views corresponding to the upper and lower walls depicted in the top two panels in Figure 1b. A similar operation can be performed for the (4,11)‐L nanotube (bottom two panels in Figure 1b). From the top view, it can be seen that the upper wall of the (11,4)‐R SWNT has the same lattice configuration as the lower wall of the (4,11)‐L nanotube (blue boxes), while the lower wall of the (11,4)‐R SWNT looks consistent with the upper wall of the (4,11)‐L SWNT (red boxes). Therefore, the configuration difference between a pair of SWNT enantiomers can also be understood as reversing the positions of the top‐view images of the upper and lower nanotube walls. Unfortunately, TEM imaging provides only 2D projective information of the 3D structure, and the object configuration along the direction parallel to the injected electron beam is lost, making it impossible to discriminate the relative positions of the upper and lower tube walls. Therefore, the top‐view atomic models, as well as the corresponding simulated TEM images and the diffraction patterns are exactly the same between the (11,4)‐R and (4,11)‐L SWNTs, demonstrating the infeasibility of distinguishing the enantiomorphic SWNTs in either real or reciprocal spaces by the simple TEM imaging approach (Figure 1c,d, Figure S2, Supporting Information). In other words, the key to resolving the SWNT handedness is to break the information degeneracy along the electron beam direction in the TEM image and to give structural information contributed from the upper and the lower nanotube walls separately.

We constructed SWNT/Gr van der Waals heterostructure with the parallel lattice orientation between the SWNT lower wall and Gr to realize the above goal. The aligned interface transforms the unsolvable problem of extracting structural information of the SWNT lower wall from the complicated moiré interference pattern in a nanotube TEM image to an easy but equivalent task of characterizing the lattice configuration of the surrounding monolayer Gr. The SWNT upper‐wall structure in real space can subsequently be obtained independently by erasing the reflexes of SWNTs that coincide with the reflexes of Gr in reciprocal space followed by performing the inverse fast Fourier transform (FFT) operation of the left reflection patterns.

**Figure 2:**
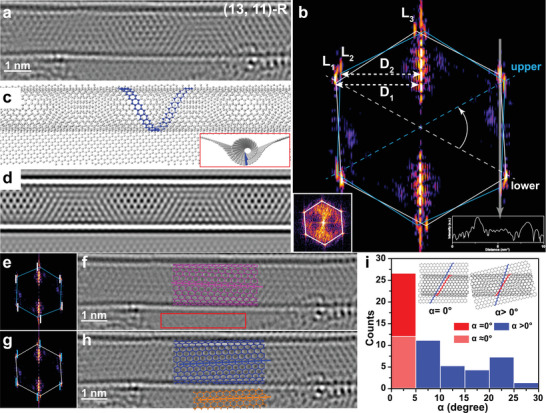
A typical example showing the identification of the SWNT handedness by one TEM image. a) TEM image of an aligned SWNT on Gr. b) FFT image of (a). Three principal layer line reflections contributed from the SWNT are labeled by *L_1_*, *L_2_*, and *L_3_*, respectively. The spacings of *L_1_* and *L_2_* with respect to the equatorial layer line are marked as *D_1_* and *D_2_*, respectively. The white hexagon labels the reflexes contributed from the SWNT lower wall and Gr, which are superimposed. The blue hexagon marks the reflexes from the SWNT upper wall. A pair of *L_1_* principal layer lines contributed by the upper SWNT is labeled by a blue dashed line, while the other pair of *L_1_* reflexes contributed by the lower SWNT wall is marked by a white dashed line. They correspond to the helix along a zigzag direction in a SWNT that adopts the minimum screw pitch, as represented by the blue spiral in (c). Inset on the bottom left corner is the reflexes from Gr in the vicinity of the SWNT. The intensity line profile on the bottom right corner is taken along the white arrow in (b). c) Projective view of the SWNT/Gr atomic model based on (a). Inset displays the DFT relaxed side view of the model with the blue lines representing two zigzag lattice directions in the SWNT lower wall and Gr, respectively, which are parallel with each other. d) TEM image simulation based on the model in (c). e) FFT image of (a) with reflexes from Gr and the SWNT lower wall removed, as labeled by the short white lines. f) Reconstructed TEM image based on the FFT image in e), in which the lattice of the SWNT lower wall and Gr is filtered, as verified by the red box. The lattice configuration of the SWNT upper wall is represented by the pink model. g) FFT image of panel a with reflection spots from the SWNT upper wall removed, as labeled by the short blue lines. h) Reconstructed TEM image based on the FFT image in (g), in which the lattice of the SWNT upper wall is filtered. Blue and orange atomic models are superimposed on the reconstructed TEM image to represent the lattice structures of the SWNT lower wall and Gr, respectively. i) Histogram showing the distribution of the stacking angles (*α*) between SWNTs and Gr.

The main preparation steps of the mixed‐dimensional SWNTs/Gr van der Waals heterostructures are schematically illustrated in Figure 1e,f (See Experimental Section). SWNTs and Gr were first grown on the insulating substrates and copper foils, respectively, by the chemical vapor deposition (CVD) methods. To verify the universality of this structural determination approach for SWNTs with different morphologies, we applied two growth strategies to produce randomly aligned SWNTs on quartz and horizontal SWNT arrays on a‐sapphire, respectively (Figure S3, Supporting Information). A polymer‐assisted transfer method was conducted to sequentially peel off SWNTs from the substrate followed by scooping up the polymer/SWNTs film floating on the deionized water using a Gr/copper (Cu) substrate. Therefore, a polymer/SWNTs/Gr/Cu vertical heterostructure with a thin layer of water trapped at the SWNT/Gr interface was generated (Figure 1e). It is worth noting that, to increase the parallel alignment of SWNTs on Gr, the water film should be dried slowly so that the lower nanotube wall can approach the Gr surface gradually, leaving sufficient relaxation time for the heterojunction to converge to the thermodynamically favorable structure with parallel alignment at the interface (Figure 1f, see details in Figure S4, Supporting Information). The high‐quality CVD‐grown graphene with a super‐clean surface,^[^ ^28^, ^29^ ^]^ as well as the annealing process of the TEM specimen before imaging, could also facilitate interfacial alignment. All these measures make the SWNT/Gr specimen with aligned carbon‐carbon interfaces account for nearly half of the total samples, which is close to the results achieved by laser irradiation cleaning in a high vacuum (see details in **Figure** **2**i).^[^ ^25^ ^]^ The copper foil was then etched away and the heterostructure was transferred to a holey TEM grid. Finally, the polymer film was dissolved and removed by acetone, leaving suspended SWNTs/Gr heterostructures for TEM imaging (Figure 1m). It is worth noting that SWNTs are on top of Gr when the TEM grid is loaded into the TEM column (Figure S5, Supporting Information). The handedness analysis introduced in Figure 2 is based on this premise.

Figure 1g‐l exhibits the evolution of the atomic structure (real space) and the diffraction pattern (reciprocal space) for the SWNT/Gr heterostructure when its interfacial configuration changes from arbitrary to parallel alignment. The random orientation of a SWNT on Gr (Figure 1g,h) results in the appearance of three groups of reflexes in the reciprocal space of the heterostructure (Figure 1i). Two groups having streaks arise from the upper and lower walls of the SWNT with helical structures (pink lines), while one group of reflexes with sharp spots originate from the Gr lattice (yellow lines). However, in this scenario, it is incapable of assigning the attribution of the two sets of SWNT reflexes to the upper and lower nanotube walls, respectively. When the SWNT lower wall is parallelly oriented with Gr (Figure 1j,k), the reflexes from Gr and from the SWNT lower wall coincide (yellow lines) with clear identification of their lattice orientation. In this case, only two groups of reflexes are present for the heterostructure (Figure 1l), and the reflexes highlighted by pink lines can be unambiguously assigned to the SWNT upper wall. These schematics demonstrate the methodology of how to separate the structural information of the SWNT upper and lower walls separately with the assistance of a single layer of Gr so that the 3D chirality information (handedness) can be restored using one TEM photograph without sample tilting.

Figure 2a‐d shows how to apply this method to identify the complete structure of a SWNT in a TEM image. In brief, there involve four steps. First, verify whether the SWNT is chiral and whether its lower wall is parallelly oriented on Gr so that objects that are applicable to this method can be confirmed. Second, assign reflexes in the FFT image that belong to the upper and lower SWNT walls separately based on the intensity asymmetry and determine the handedness. Third, identify several possible chiral indices (*n,m*) of the SWNT based on the measurement of the principle line spacings in the FFT image combined with the diameter measurement in the TEM image.^[^ ^26^ ^]^ Finally, perform TEM image simulation corresponding to the atomic models of heterostructures with all possible SWNT chiral indices and determine the most fitted (*n,m*) value according to the matching degree between the experimental and the simulated images.^[^ ^27^ ^]^

Figure 2a is the TEM image of a SWNT/Gr heterostructure with its corresponding FFT image displayed in Figure 2b. Two groups of reflexes are seen, both of which show streaks elongated along the directions perpendicular to the tubule axis, indicating their origins from the upper and lower walls of a chiral SWNT. The intensity line profile measured along the white arrow unveils that the brightness of the former streak is higher than that of the latter one (inset on the bottom right corner in Figure 2b). Therefore, the bright streaks connected by the white hexagon can be assigned to the SWNT lower wall and Gr with parallel orientation, while the dim reflexes are contributed solely from the SWNT upper wall. We then connected the first principal layer line reflexes (*L_1_*) that are contributed from the SWNT upper wall (a blue dashed line) and lower wall (a white dashed line), respectively. It shows that the white line needs to be rotated counterclockwise with a twist angle of <60° to turn into the blue line, indicating that the spiral along a zigzag lattice direction with the smallest screw pitch is right‐handed (Figure S6, Supporting Information). Therefore, based on the handedness definition of a SWNT,^[^ ^30^ ^]^ this (13, 11) nanotube is right‐handed.

The ratio of the chiral indices *m*/*n* was obtained by the following equation^[^ ^26^ ^]^

(1) $$\frac{m}{n}=\frac{2D_{2}-D_{1}}{2D_{1}-D_{2}}$$

where *D_1_* and *D_2_* are the spacings from the principal layer lines *L_1_* and *L_2_* to the equatorial line, respectively (Figure 2b). The chiral indices ratio is 0.848 with the nanotube diameter measured in (a) to be ≈1.6 nm. By looking up the table,^[^ ^26^ ^]^ three values of (*n,m*) are possible due to the measurement error, which are (12,10), (13,11) and (14,12), respectively. Image simulations were conducted based on the DFT‐modified atomic models of the heterostructures with different SWNT chiral indices. The moiré pattern from the simulated TEM image of a (13,11) SWNT agrees with the experimental image best (Figure 2c,d). Therefore, the complete structure of the SWNT in Figure 2a is (13,11)‐R. Figure 2e‐h provides a real‐space handedness identification method with equivalent results by separately reconstructing the atomic structures corresponding to the SWNT upper and lower walls. By performing the inverse FFT to the reflection pattern after removing one set of reflexes marked by the short white lines (Figure 2e), the lattice configuration belongs to the SWNT upper wall is independently identified with structural information from the SWNT lower wall and Gr filtered (red box). Similar operations can be implemented to independently unveil the atomic structure of the SWNT lower wall and Gr (Figure 2g,h), which certifies the parallel alignment between Gr and the SWNT lower wall (blue and yellow atomic models in Figure 2h) and intuitively discloses the atomic structures of the SWNT upper and lower walls separately (the pink model in Figure 2f and the blue model in Figure 2h), double affirming the right‐asv=2 handedness of this SWNT.

Thanks to the high spatial resolution of TEM imaging, this strategy is not only applicable to the isolated SWNT but can also distinguish the full structure of SWNTs locating several nanometers apart tube by tube as long as they are well aligned on Gr (Figure S7, Supporting Information). It is worth noting the probability that all SWNTs in a bundle were oriented with Gr was found to be relatively low, because, in this scenario, the factors affecting the nanotube orientation are not only limited to the Gr underneath but also include the surrounding SWNTs (Figure S8, Supporting Information). We systematically analyzed 55 distinct interfaces between SWNTs and Gr whose lattice orientations can be unambiguously identified and plotted the stacking angle (*α*) distribution in Figure 2i (Figure S9, Supporting Information). 49.1% of SWNTs prefer the parallel alignment with Gr having misorientation angles less than 5° (although we cannot distinguish which set of reflexes correspond to the upper SWNT wall and which group of reflexes arise from the lower tube wall) (Figure 2i). The results deviate significantly from the random distribution, indicating the energetic advantage of the aligned SWNT/Gr interface compared with the turbostratic stacking, consistent with the previous work.^[^ ^25^ ^]^ The result also illustrates the feasibility of this Gr‐assisted complete structural determination method due to the high propensity of parallel stacking achievement.

### 2.2 Interfacial Configuration of SWNT/Gr van der Waals Heterostructures

**Figure 3:**
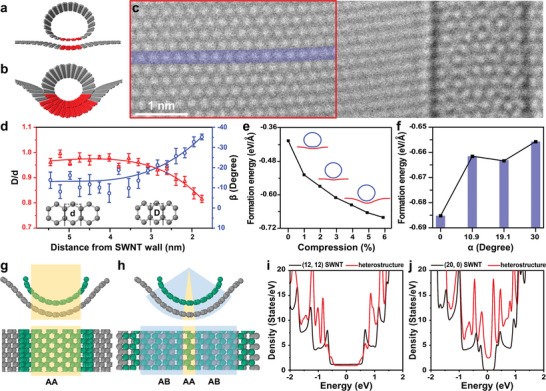
Interfacial configuration of SWNT/Gr van der Waals heterostructures. a,b) 3D perspective atomic models representing a “line contact” mode and a “surface contact” mode, respectively, between Gr and the SWNT lower wall. The contact areas at the interface are highlighted in red. c) TEM image showing prominent lattice distortion of Gr when it approaches a SWNT. d) Plot revealing the projective interatomic distance shrinkage ratio of the graphene lattice as a function of the distance from the SWNT left wall (red triangles). The interatomic distance represented in the inset is measured in the red‐boxed region in (b) along the purple line direction from left to right. The intrinsic interatomic distance in a benzene ring of Gr is represented as *d*, while the deformed interatomic distance due to out‐of‐plane distortion is denoted as *D*. The inclination angles to the horizontal of the corresponding benzene rings are plotted as the blue circles. e) Formation energy of the (12,12) SWNT/Gr heterostructure as a function of the compressive strain of Gr, which determines the out‐of‐plane distortion of the Gr at the interface. f) Formation energy of the SWNT/Gr heterostructure as a function of the stacking angle (*α*) between the SWNT lower wall and Gr. The compression ratio of Gr in this case is 6%. g) Parallel projection showing the interface configuration of a DFT‐relaxed (13,11)‐R SWNT/Gr heterostructure with a “surface contact” mode, in which a large area at the interface displays an AA stacking configuration (yellow‐shaded region). h) Central projection of the (13,11)‐R SWNT/Gr heterostructure interface, where most areas adopt staggered stacking (close to AB stacking, blue‐shaded region) with only a small region having AA stack (yellow‐shaded region). i) DFT‐calculated density of states of an intrinsic metallic (12,12) SWNT (black line) and a (12, 12) SWNT/Gr heterostructure in a “surface contact” mode (red line), respectively. j) DFT‐calculated density of states of an intrinsic semiconducting (20, 0) SWNT (black line) and the (20, 0) SWNT/Gr heterostructure in a “surface contact” mode (red line), respectively.

We investigated the interfacial configuration of the heterostructure at the atomic scale to unveil the reason behind the alignment preference between the SWNT lower wall and Gr. There exist two possible modes. One is denoted as the “line contact” mode, in which only a few columns of carbon atoms at the SWNT bottom wall are in contact with the almost undeformed Gr surface (**Figure** **3**a). The other is defined as the “surface contact” mode, in which Gr at the interface partially folds around the curved SWNT lower wall to construct a non‐Euclidean contact surface (Figure 3b). Figure 3c is a TEM image of a SWNT/Gr heterostructure with sufficient signal‐to‐noise ratio and high surface cleanness, which are prerequisites to interpreting the out‐of‐plane deformation of Gr at the interface. We measured the intermolecular spacing deviation of Gr along the zigzag lattice orientation in the vicinity of the SWNT (the red‐boxed region in Figure 3c). If Gr partially wraps around the SWNT bottom wall and induces out‐of‐plane deformation, the intermolecular spacing of the benzene ring will be shrunk compared with the intrinsic horizontal lattice in normal projection (inset of Figure 3d), which can be quantitatively described by the lattice compression ratio (*D*/*d*). The inclination angle (*β*) of Gr relative to the horizontal plane can be derived by the formula of *β* = arccos (*D*/*d*). Figure 3d is a plot displaying the interatomic spacing compression ratio (*D/d*) in a top‐view image and the corresponding local inclination angle (*β*) of Gr as a function of the distance from the SWNT wall, respectively. The projective interatomic distance shrinks prominently when the distance from the nanotube wall is <3 nm. The compression ratio (*D/d*) reaches ≈0.83 when the distance is ≈1.5 nm, corresponding to an inclination angle of ～‐35°. Further approaching the SWNT gives rise to severe out‐of‐plane deformation, which results in a large defocus deviation of the local Gr from Scherzer defocus, thus inducing a blurry lattice (Figures S10, S11, and S12, Supporting Information). These results demonstrate the “surface contact” mode at the heterostructure interface where Gr generates a long‐range linear groove to partially embed the SWNT. The maximized contact area and the curved interfacial morphology could be derived from the strong interlayer interaction between the *sp^2^*‐hybridzied carbon nanomaterials, the high flexibility of the suspended 2D Gr sheet, as well as the slow drying and annealing processes in the sample preparation.

Molecular dynamic simulations were performed to understand the interfacial contact mode. Figure 3e shows the variation in the formation energy of the heterostructure as the interfacial morphology evolves from the “line contact” to the “surface contact”. By gradually increasing the compressive strain in Gr perpendicular to the SWNT tube axis, the contact area at the interface between SWNT and Gr expands. The formation energy of the heterostructure decreases monotonically by ≈280 meV Å^−1^ as the compressive strain increases from 0 to 6%, indicating the energetic advantage of the “surface contact” mode. The effect of the stacking angle (*α*) between SWNT and Gr on the formation energy of the heterostructure at a compressive strain of 6% was shown in Figure 3f, and an obvious thermodynamic advantage of the parallel alignment over other stacking angles is clearly displayed. These results well support our experimental observation of the abnormally high distribution of the aligned interfacial configuration in SWNTs/ Gr heterostructures.

We also investigated the interlayer atomic registries at the curved heterostructure interface based on the DFT‐optimized atomic model of the (13,11)‐R SWNT/Gr shown in Figure 2c. In bilayer Gr with flat morphology, AB stacking is the most energetically favorable,^[^ ^31^, ^32^ ^]^ in which half of the atoms in one layer situate directly on top of the hexagonal ring centers of the other layer. In contrast, AA stacking, where two layers are exactly aligned, is less common. Surprisingly, the parallel projection view of the DFT‐relaxed (13,11)‐R SWNT/Gr heterostructure displays an AA‐stacked configuration (Figure 3g). We then wrote code to unveil a central projection image of the curved heterojunction interface (Figure 3h), which ensures that every line of sight emanating from the tube center is perpendicular to the SWNT wall, thus displaying a real projective view of the curved heterostructure interface. In this case, it shows that AA stacking only exists at the nanotube bottom with two columns of atoms wide (yellow‐shaded region). Then, the interlayer registries quickly evolve to the near‐AB stacking with the staggered atomic arrangement as the location progresses further to both sides of the nanotube. A similar stacking phenomenon also exists when changing a chiral nanotube to an armchair one on Gr (Figure S13 and S14, Supporting Information). These results indicate an increased complexity and diversity of the interlayer stacking due to the presence of curvature at the *sp^2^*‐hybridized carbon‐carbon interface.

To unveil the impact of the Gr addition on the evolvement of the SWNT electronic structures, we chose a metallic (12,12) SWNT and a semiconducting (20, 0) SWNT as representatives, whose tube diameters are similar to the (13, 11)‐R SWNT observed in our experiment and calculated their density of states (DOS) before and after generating the van der Waals heterostructure with Gr in a “surface contact” mode with parallel interfacial stacking. The difficulty of directly calculating the electronic properties of chiral SWNT/Gr heterostructures lies in the large size of the unit cells and the accompanying huge computational effort. Figure 3i shows that the (12,12) SWNT/Gr heterostructure remains metallic, consistent with the intrinsic (12, 12) SWNT. However, the semiconducting (20, 0) SWNT turns into conducting after forming the (20, 0) SWNT/Gr heterostructure (Figure 3i). In addition, the van Hoff singularities of both two one‐dimensional (1D) heterostructures are significantly disparate from those of the pristine SWNTs due to the interlayer interaction between the SWNT lower wall and Gr with a 0° stacking angle. The unique electronic properties of these 1D heterostructures can be observed by Raman, photoluminescence, or light absorption experiments^[^ ^33^, ^34^ ^]^ and indicate the diversified effect of Gr on the electronic modulation of SWNTs.

**Figure 4:**
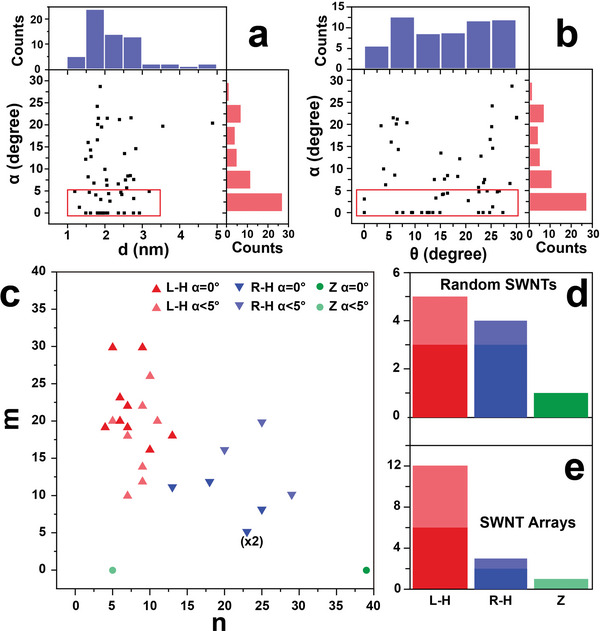
Generalizability of the Gr‐assisted SWNT handedness determination method and the handedness distributions for two types of SWNTs. a) Plot of the stacking angle (*α*) versus the SWNT diameter (*d*). The red‐boxed region highlights the diameter distribution of those SWNTs that are aligned with Gr with stacking angles less than 5°. b) Plot of the stacking angle (*α*) versus the SWNT helical angle (*θ*). The red‐boxed region highlights the helical angle distribution of those SWNTs that are aligned with Gr with stacking angles less than 5°. c) Plot showing the detailed chiral indices and handedness of 27 SWNTs, which adopt two types of morphologies due to different growth methods. R‐H, L‐H, and Z represent right‐handed, left‐handed, and zigzag SWNTs, respectively. SWNTs that are perfectly aligned with Gr (*α* = 0°) are represented by red, blue, and green markers without transparency, while SWNTs that are quasi‐parallelly stacked on Gr (*α* < 5°) are represented by red, blue, and green markers with 50% transparency. d) Histogram showing the handedness distribution of randomly aligned SWNTs grown on ST‐cut quartz, revealing similar proportions of left‐ and right‐handed nanotubes. Columns in nontransparent colors represent SWNTs that are perfectly aligned with Gr, while columns in semi‐transparent colors represent SWNTs that are quasi‐parallel with Gr (*α* < 5°). e) Histogram showing the handedness distribution of horizontal SWNT arrays grown on a‐sapphire, implying potential left‐handedness enrichment.

**Figure** **4**a plots the correlation between the stacking angle (*α*) of the SWNT/Gr heterostructures and the SWNT diameter (*d*). 55 specimens were examined, showing SWNT diameters dominantly ranging from 1 to 3 nm. The plot of the stacking angle (*α*) versus the SWNT helical angle (*θ*) in Figure 4b displays the near‐uniform distribution of the SWNT helical angles for the examined nanotubes, indicating that the sampling is extensive. We do not detect significant data agglomeration in either scatterplot, implying no obvious dependence of the stacking angle on a particular diameter or helical angle. Data points in the red‐boxed regions representing the SWNTs that are parallelly stacked on Gr with misorientation less than 5° also show a wide and discrete distribution in the dominant value range of the SWNT diameter and helical angle corresponding to the total sample. These results suggest that the formation of the parallelly stacked interface in heterostructures is independent of both the diameter and the helical angle of SWNTs, indicating the generality of this Gr‐assisted handedness determination strategy for SWNTs with diversified morphologies.

We plotted the complete structures, including the chiral indices and the handedness, of 27 SWNTs synthesized by two distinctive approaches to establish the relationship between the SWNT handedness distribution and the preparation methods (Figure 4c).15 nanotubes are perfectly aligned with Gr, while 12 nanotubes are quasi‐parallel with Gr with stacking angles (*α*) less than 5°, which are also included in the handedness analysis to expand the sample pool. Figure 4d,e summarizes the handedness distribution of randomly orientated SWNTs grown on ST‐cut quartz and horizontally aligned SWNT arrays grown on a‐sapphire, respectively. No handedness priority was observed in randomly oriented SWNTs. The absence of enrichment may arise from two reasons: (1) the usage of copper nanoparticles with a low melting point as catalysts, which transformed into the liquid phase at the SWNT growth temperature of ～850°C, thus lacking the ability to regulate the SWNT configuration; (2) the negligible template effect of the quartz substrate, which resulted in the unaligned assembly of SWNTs, as well as the absence of handedness selectivity of the product, similar to the phenomena reported for SWNTs achieved in the vapor phase by the kite‐flying method.^[^ ^19^, ^21^ ^]^ Interestingly, horizontal SWNTs arrays grown on a‐sapphire exhibit a formation preference for left‐handed nanotubes. When only considering SWNTs in perfectly aligned heterostructures, 6 SWNTs over 8 samples investigated are left‐handed, yielding a ratio between left‐ and right‐handed SWNTs of 3:1. If expanding the data pool to quasi‐aligned SWNT/Gr heterostructures, 12 SWNTs over 15 chiral tubes are left‐handed, whose number significantly deviates from the random distribution. This is therefore suggestive that selective formation of handed nanotubes may occur in the horizontal SWNT arrays prepared on the (110) lattice plane of sapphire. Potential reasons may stem from the absence of mirror symmetry of the intrinsic a‐sapphire substrate. Moreover, the complex atomic kinks on the sapphire surface reconstructed during the high‐temperature annealing, as well as the adsorption of catalyst nanoparticles at special atomic sites, could further decrease the surface symmetry of the substrate. They may empower (110) crystal facets of sapphire with surface chirality^[^ ^35^, ^36^ ^]^ and hence lead to the selective growth of left‐handed SWNTs. However, further investigations are needed including expanding the sample size and developing novel methodologies to verify our observations via disparate mechanisms. The electrical property analysis of SWNTs grown by two different CVD methods based on the chiral indices has also been investigated (Table S1, Supporting Information).

## 3 Conclusion

In summary, a simple, swift, and reliable method that can determine the complete structure of SWNTs, especially handedness, at the atomic level by one TEM image has been developed. It has simple requirements on equipment, operation, sample preparation, and data analysis, which is accessible to most synthesis groups and can seamlessly couple with the preparation experiments. The method makes use of the construction of SWNTs/Gr van der Waals heterostructures with aligned interfaces to realize the lattice interpretation of SWNT upper and lower walls separately, thus transforming the 3D handedness information to projected 2D space. The parallel stack between SWNTs and Gr at the interface is energetically preferred with Gr displaying prominent out‐of‐plane deformation to maximize the contact area with the curved SWNT lower walls. The construction of the aligned heterostructures shows no prominent sensitivity to the SWNT helical angle and diameter. It also does not make special requests on the sample morphology and preparation approaches. This handedness identification strategy raises an idea showing how to break the structural information degeneracy of nanomaterials like SWNTs along the electron beam direction in TEM imaging with the assistance of an aligned 2D sheet as media. The method can be extended to the chirality determination of other 1D tubular enantiomorphic structures or 2D atomic thin films with twist angles, facilitating both the fundamental exploration and practical applications of chiral physics and chiral devices in low‐dimensional materials. The inequivalent distribution of left‐ and right‐handed SWNTs in the horizontal arrays grown on a‐sapphire may expand the dimension of controlled SWNT growth from electronic properties and chiral indices to handedness that may inspire further insights into the effect of substrates and solid catalysts with asymmetric surfaces.

## 4 Experimental Section

### CVD Synthesis of SWNTs

The randomly oriented SWNT networks were synthesized on the ST‐cut quartz substrate (single‐side polished, miscut angle < 0.5°, surface roughness < 5 Å, Hefei Kejing Materials Technology Co., China), which underwent an annealing process at 900 °C in the air for 8 h before the SWNT growth. A piece of copper coil was put under the quartz substrate and heated in argon to 850 °C. After the system was purged with 300 standard cubic centimeters per minute (sccm) argon and 300 sccm hydrogen, a 40 sccm of argon (through an ethanol bubbler) was flushed out to grow SWNT for 20 min.

The horizontally aligned SWNT arrays were synthesized by CVD using Trojan‐Mo catalysts similar to the previous work.^[^ ^16^, ^37^ ^]^ Fe catalysts (0.05 mM Fe(NO_3_)_3_/ethanol solution) were spin‐coated on the a‐plane sapphire substrate (Hefei Kejing Materials Technology Co., China) followed by an annealing process at 1100 °C for 8 h in air. Then, Mo catalysts (0.05 mM (NH_4_)_2_MoO_4_/ethanol solution) were loaded on the substrate by spin coating. During the growth, the pretreated substrate was first heated in air at 850 °C for 15 min. The system was then purged by argon with a flow rate of 300 sccm for 5 min. Finally, 200 sccm hydrogen and 50 sccm argon were introduced through an ethanol bubbler into the system with the substrate maintained at 850 °C for 30 min, leading to the growth of SWNT horizontal arrays.

### Preparation of the SWNTs/Gr Van Der Waals Heterostructures

A thin film of poly (methyl methacrylate) (PMMA) was initially spin‐coated on the SWNT/quartz or SWNT/sapphire substrate surface. Subsequently, the sample was gently floated on a 1 mol L^−1^ potassium hydroxide (KOH) solution heated at 100 °C to etch quartz or sapphire away. When the PMMA/SWNT film detached from the substrate, the film was transferred to the deionized water for three times to thoroughly remove residuals left by the etchant. The PMMA/SWNTs film then was scooped up by monolayer Gr grown on the copper foil to form PMMA/SWNTs/Gr/Cu heterostructure. The residual water at the SWNTs/Gr interface was evaporated slowly by either natural drying in the air or baking with the infrared light at a mild temperature so that the bottom surface of SWNT can approach Gr gradually, which is conducive to the epitaxial alignment of SWNTs on Gr. The copper foil was etched away in the 1 M solution. The PMMA/SWNTs/Gr vertical heterostructure film was thoroughly rinsed with deionized water and scooped up by a holey silicon nitride (SiN*_x_*) TEM grid. It was dried naturally in the air and baked on the hotplate at 180 °C for 15 min. The PMMA scaffold was finally removed by submerging the TEM grid in acetone for 8 h. The specimen was annealed in the mixed argon/hydrogen atmosphere at 300 °C before TEM imaging.

### Aberration‐Corrected Transmission Electron Microscopy and Image Processing

The high‐resolution TEM was conducted using Titan Cubed Themis G2 300 equipped with probe and image aberration correctors under an accelerating voltage of 80 kV. TEM images were recorded using a Gatan OneView CCD camera with a 2–4 s acquisition time. The pixel resolution for each TEM image is 170 pixels nm^−1^. The electron dose used for imaging was ≈0.07 pA nm^−2^. Images were processed using ImageJ software. They were initially adjusted with a band‐pass filter (between 100 and 1 pixels) to modify the long‐range nonuniformity on the illumination intensity and then smoothed by applying a Gaussian blur (2−4 pixels). Atomic models were established via the software of Material Studio and optimized by MD simulation. Simulated multislice images based on corresponding atomic models were generated using QSTEM software with a proper parameter adjustment according to the TEM experimental condition.

### MD Calculation

An in‐house code, METCAR, was employed to run the molecular dynamics (MD) simulations. The intra‐ and interlayer interactions between carbon atoms were described by the second‐generation reactive empirical bond order (REBO) potential^[^ ^38^ ^]^ and a modified Lenard‐Jones potential, respectively. During the MD simulations, Newton's equation of atom's motion was integrated by the Velocity Verlet algorithm and a Nose‐Hoover^[^ ^39^ ^]^ thermostat was applied to maintain the temperature of the system at 1 K. The (13,11) SWNT was aligned on the top of a Gr layer with the same chiral and wrapped by compressing the Gr layer. The initial supercell volume of this system was modeled as 228.92 × 89.59 × 100 Å^3^, and the MD simulations were run 100 ps per 1% of compressive strain up to 6%. The periodical boundary condition was applied, and the total number of atoms is 10 392, including 1732 atoms in the (13,11) SWNT and 8660 in the Gr. The time step was 1 fs. To validate that the SWNT/Gr heterojunction structure with the same chiral orientation has the lowest energy, the MD simulations of a (12,12) SWNT aligned on a Gr sheet with the rotation angle of 0°, 10.9°, 19.1°, and 30° were carried out to explore their wrapping processes under different compression conditions. Based on the symmetry of Gr, only the rotation angles between 0° and 30° were considered here. The formation energies of the (12,12) SWNT atop the graphene sheet with rotation angles of 0°, 10.9°, 19.1°, and 30° were obtained by subtracting the energies of the uncompressed Gr and (12,12) SWNT from the overall energies of the stabilized SWNT/Gr heterostructures.

### DFT Calculation

The density of states (DOS) of the intrinsic graphene, intrinsic (12,12) and (20,0) SWNTs, and their SWNT/Gr heterojunctions were revealed by density functional theory (DFT) calculations using the Vienna Ab Initio Simulation Package (VASP).^[^ ^40^ ^]^ The generalized gradient approximation in the Perdew–Burke–Ernzerhof functional^[^ ^41^ ^]^ is applied, and the cutoff energy of the plane wave is set as 400 eV. The convergence criterion for energy and force was set to 1e^−4^ eV and 0.01 eV Å^−1^. The vacuum layer was larger than 10 Å to avoid interactions of neighboring images. The van der Waals (vdW) interaction between the SWNT and Gr was described by the DFT‐D2 method of Grimme.^[^ ^42^ ^]^ For the (12,12) SWNT/Gr and (20,0) SWNT/Gr heterostructures, a 5 × 5 × 1 grid was used to relax the whole structure, the DOS of which was obtained by using 50 × 1 × 1 and 20 × 1 × 1 grids to calculate the bandgap, respectively. For the graphene, a 5 × 5 × 1 grid was used in structural relaxation, and a 39 × 39× 1 grid was used to calculate the DOS. For (12,12) and (20,0) CNT, a 1 × 1 × 5 grid was used in structural relaxation, and a 1 × 1 × 80 and a 1 × 1 × 30 grid were used to calculate the bandgap, respectively.

## Conflict of Interest

The authors declare no conflict of interest.

## Author Contributions

Y.Y., Y.Z., and S.Li contributed equally to this work. J.Z. and S.W. initiated the project and generated the experimental protocols. Y.Y., S. Li, and S.W. fabricated the STEM sample and conducted ADF‐STEM imaging. Y.Z., C.Z., and F.D. performed DFT calculations. W.L. prepared SWNTs. All authors contributed to the data analysis, manuscript writing, and revision of the manuscript.

## Supporting information

Supporting Information

## Data Availability

The data that support the findings of this study are available from the corresponding author upon reasonable request.
