# Supplementary material for: Determine the Complete Configuration of Single‐Walled Carbon Nanotubes by One Photograph of Transmission Electron Microscopy
Source: Adv Sci (Weinh). 2023 Mar 25;10(15):2206403. doi: 10.1002/advs.202206403 (PMC10214254; doi:10.1002/advs.202206403)
Supplement: Supplementary file 1 — Supporting Information [file ADVS-10-2206403-s001.pdf]

Supporting Information

Determine the complete configuration of single-walled carbon nanotubes by one photograph of transmission electron microscopy

Yue Yu, Yifan Zhao, Shouheng Li, Chao Zhao, Weiming Liu, Shanshan Wang, Feng Ding,* and Jin Zhang**

This PDF file includes:

Figure S1. Definitions depicting left- and right-handedness of a helix and a chiral SWNT, respectively.

Figure S2. Infeasibility of distinguishing the handedness of SWNTs by the simple TEM imaging approach.

Figure S3. Synthesis results of horizontal SWNT arrays and randomly aligned SWNTs, respectively.

Figure S4. Fabrication of SWNTs/Gr heterostructures.

Figure S5. Relative position between SWNTs and Gr in the TEM column

Figure S6. Determining the handedness of SWNT in reciprocal space.

Figure S7. Determining the full structure of SWNTs locating closely to each other tube by tube.

Figure S8. SWNT alignment in a bundle.

Figure S9. Determination of the stacking angle (α) in a SWNT/Gr heterostructure.

Figure S10. Out-of-plane deformation of Gr close to SWNTs.

Figure S11. Simulated AC-TEM images corresponding to Gr with different types of out-of-plane distortion.

Figure S12. AC-TEM image showing the Gr lattice bending on both sides of a SWNT.

Figure S13. Interfacial stacking configurations of (12,12) SWNT/Gr van der Waals heterostructures.

Figure S14. Interfacial stacking models of (12,12) SWNT/Gr heterostructure and (13,11)-R SWNT/Gr heterostructure under different strains of graphene.

Table S1. Electrical property classification of SWNTs based on their chiral indices.

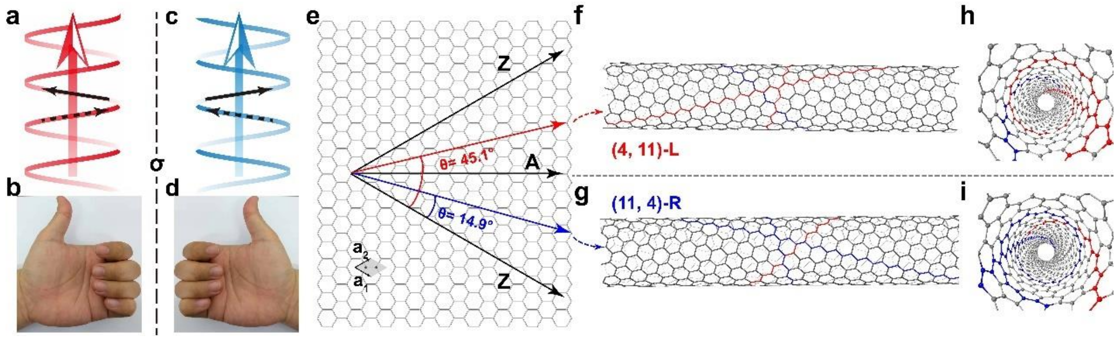

Figure S1. Definitions depicting left- and right-handedness of a helix and a chiral SWNT, respectively. (a-d) Schematics displaying a left-handed helix (red) and a right-handed helix (blue), respectively. The semi-transparent color (marked by black solid lines) represents the half wall of the helix that is closer to the viewer, while the non-transparent color (marked by black dashed lines) represents the other half wall of the helix that is farther away from the viewer. (e) Schematic diagram of rolling up a single-layer graphene into a seamless cylinder to form (f) a (4,11)-L SWNT and (g) a (11,4)-R SWNT, respectively. (h) Perspective view of a (4,11)-L SWNT along the tube axis. (h) Perspective view of a (11,4)-R SWNT along the nanotube axis. The left-handed zigzag helices in the SWNT are marked in red, while the right-handed zigzag helices are marked in blue. The SWNT handedness is defined by the handedness of most of helices along the zigzag lattice directions in a SWNT[1]. If two of the three helices along the zigzag directions are left-handed, the SWNT is defined as left-handed. In contrast, if two of the three helices along the zigzag directions are right-handed, the SWNT is defined as right-handed. In other words, the handedness of a SWNT can also be determined by the handedness of the zigzag helix that adopts the minimum screw pitch. The determination results based on these two criteria are the same.

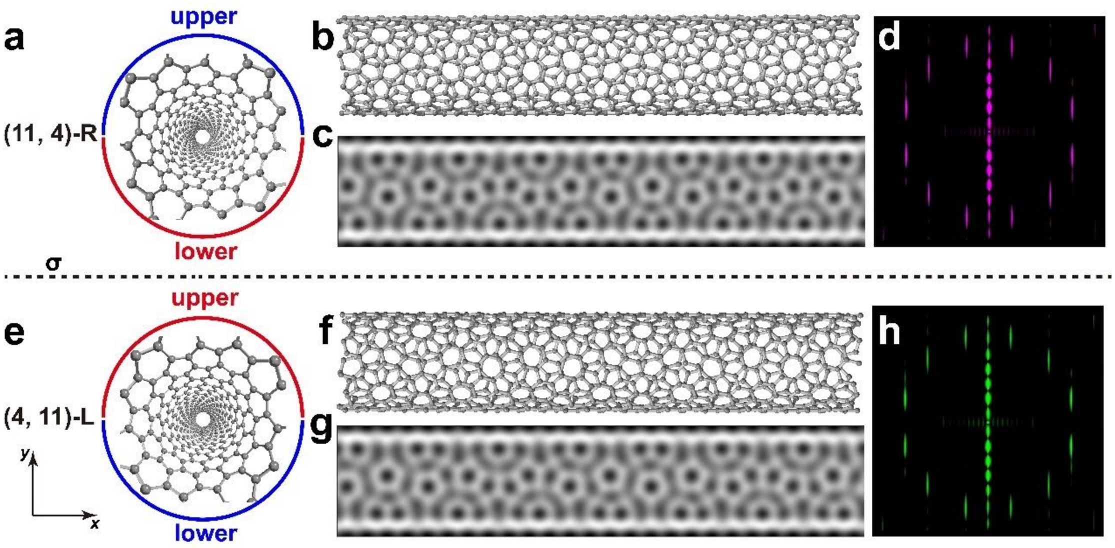

Figure S2. Infeasibility of distinguishing the handedness of SWNTs by the simple TEM imaging approach. (a, e) Atomic models of a (11, 4)-R and a (4, 11)-L SWNTs seen along the nanotube axes, respectively. (b, f) Atomic models of the (11, 4)-R and a (4, 11)-L SWNTs seen along the electron beam direction, respectively. (c, g) Simulated AC-TEM images based on the atomic models in (b) and (f), respectively, based on which it is impossible to distinguish the left- and right-handedness. (d, h) Simulated diffraction patterns based on the models in (a) and (b), respectively, which still does not display observable discrepancy between two enantiomorphic SWNTs.

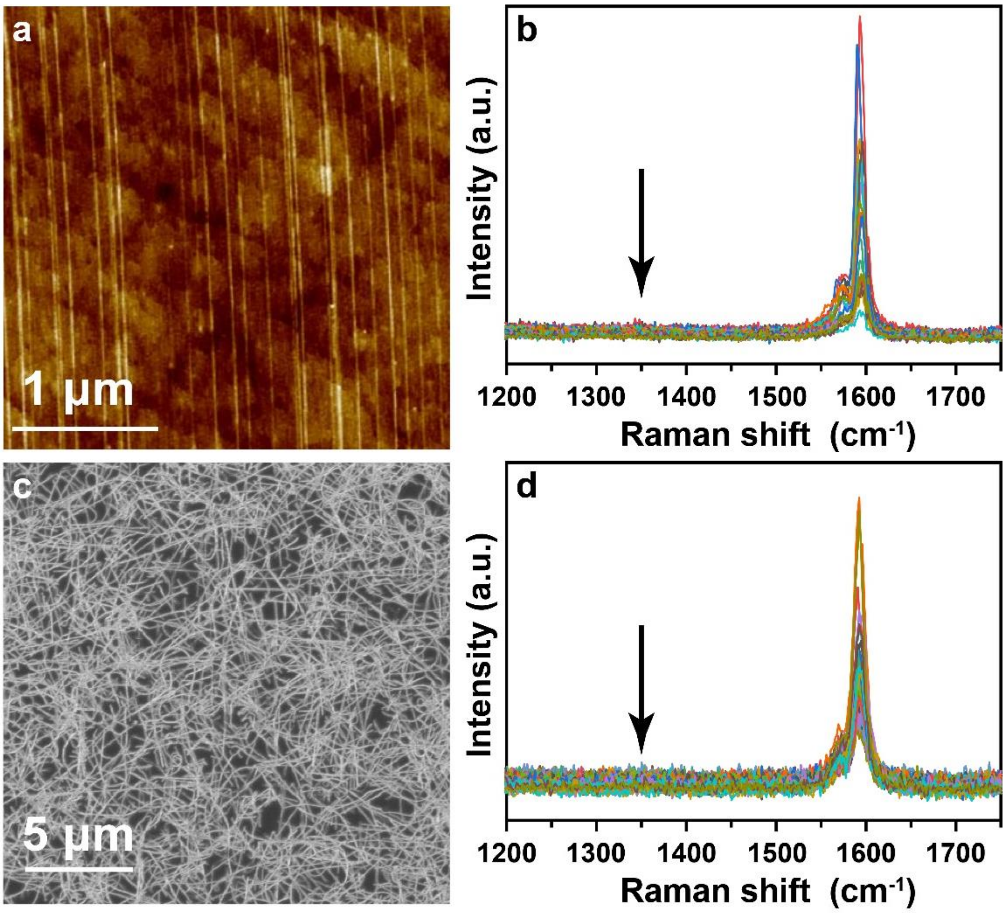

Figure S3. Synthesis results of horizontal SWNT arrays and randomly aligned SWNTs, respectively. (a, b) AFM image and the Raman spectra of the horizontal SWNT arrays grown on the a-sapphire substrate, respectively. (c, d) SEM image and the Raman spectra of the randomly aligned SWNT grown on the quartz substrate, respectively. The black arrows marked in the Raman spectra in (b) and (d) indicate the negligible D peak located at ～1350 cm^{-1}, demonstrating the high crystallinity of SWNTs.

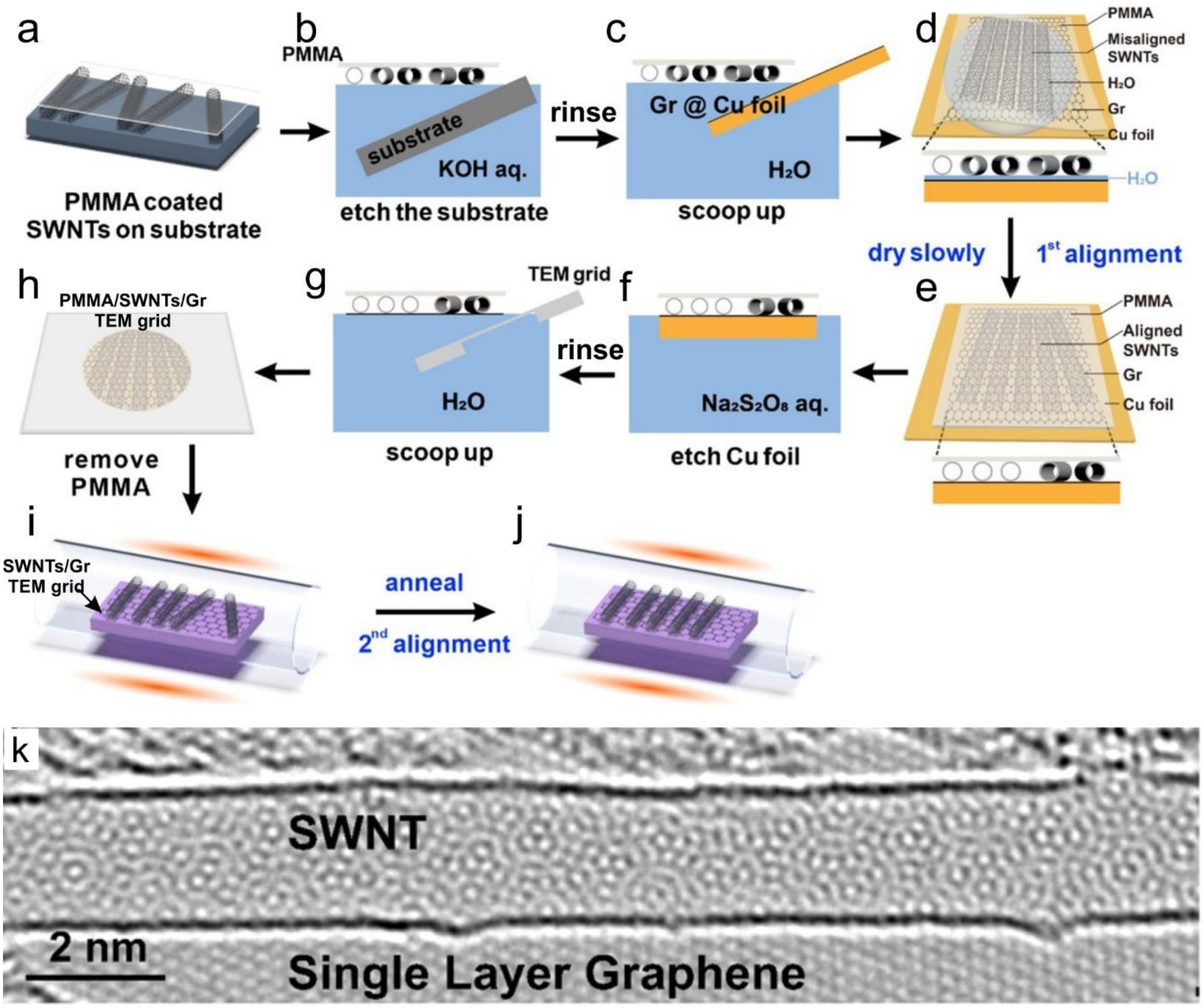

Figure S4. Fabrication of SWNTs/Gr heterostructures. (a-i) Schematic illustration displaying the detailed process of fabrication the SWNTs/Gr van der Waals heterostructures on a holey TEM grid. (k) AC-TEM image of the suspended SWNT/Gr heterostructure.

The alignment between SWNTs and Gr is expected to mainly occur in two stages. One is when the CVD-grown SWNTs covered with a thin film of PMMA is transferred to Gr and the interfacial water is slowly dried (Stage 1, Figure S4d,e). The other stage is when the SWNTs/Gr TEM sample is annealed before TEM imaging (Stage 2, Figure S4i, j). In Stage 1, after scooping the PMMA/SWNTs film onto Gr grown on copper, there exists a thin film of water between SWNTs and Gr. We slowly dried the sample in the air, making the interfacial water film become thinner and thinner. Therefore, the lower walls of SWNTs can approach and align with Gr gradually due to strong carbon-carbon interaction. It is worth noting that,

although the polymer transfer method needs to spin-coat a thin PMMA membrane on the CVD-grown SWNTs sample at the initial stage, the lower walls of SWNTs are not encapsulated by the polymer (Figure S4a-c). Therefore, the SWNT low wall lattice can directly interact with Gr when PMMA/SWNTs is transferred to the Gr/copper substrate and tends to relax to the structure which is the most energetically favorable (aligned SWNT/Gr). However, since there is a thin PMMA film covered on SWNTs, it may hinder the rotation freedom of SWNTs on Gr to some extent. This disadvantage can be diminished by adding an annealing process (300°C) to the SWNTs/Gr TEM sample before imaging (Stage 2, Figure S4i, j). At this stage, the PMMA film has been removed by acetone and the thermal energy from the heating process can facilitate further alignment between SWNTs and Gr. A similar effect has been reported by Meyer et al., who applied laser treatment to the sample to help SWNT alignment on Gr.[2]

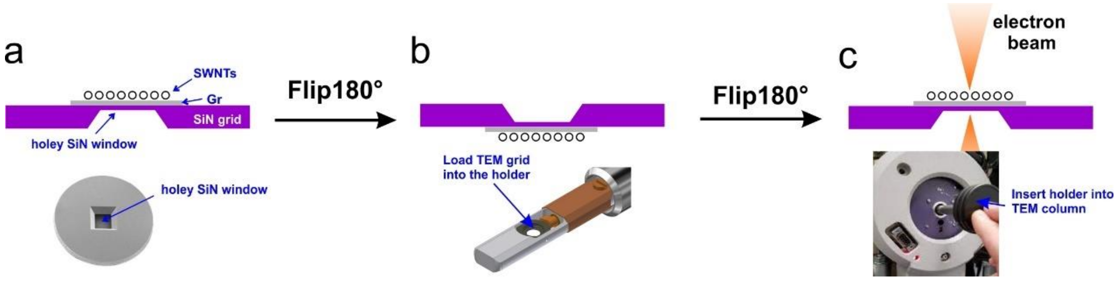

Figure S5. The relative position between SWNTs and Gr at three important experimental stages, which are the TEM sample preparation stage (a), the stage when the TEM grid is loaded into the TEM holder (b), and the stage when the TEM holder is inserted into the TEM column (c).

At the TEM sample preparation stage, SWNTs are on top of Gr (Figure S5a). Detailed transfer process has been demonstrated in Figure S4. Then, the SiN TEM grid is loaded into the holder (Figure S5b). At this stage, the TEM grid is flipped 180 degrees so that Gr is on top of SWNTs. Finally, the holder is inserted into the TEM column (Figure S5c). Since the holder needs to be rotated 180 degrees while inserting into the equipment, SWNTs are on top of Gr again with respect to the TEM.

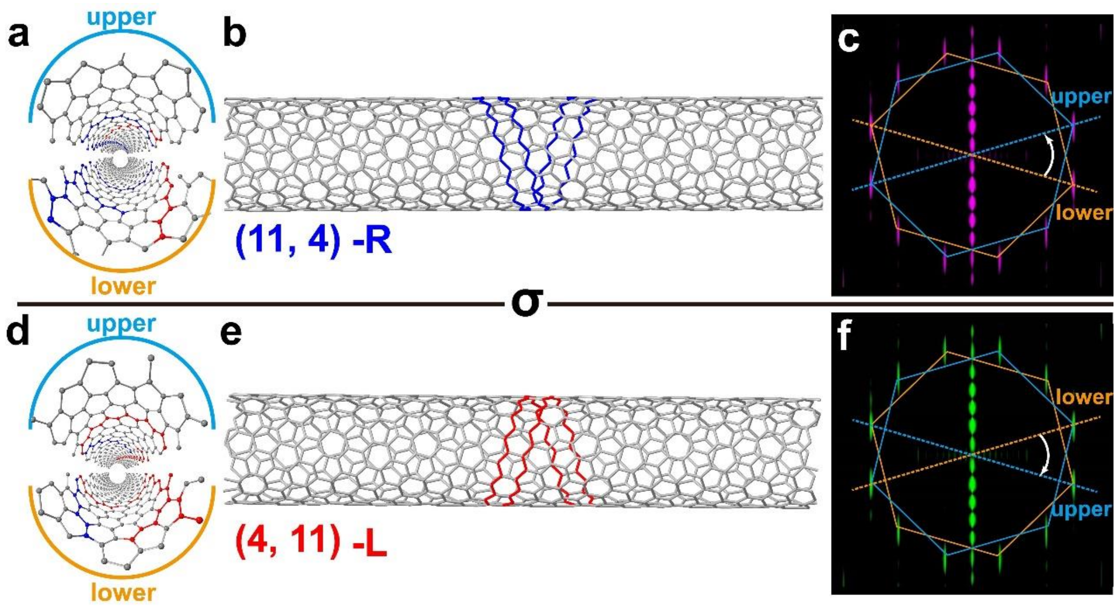

Figure S6. Determining the handedness of SWNT in reciprocal space. (a-c) Side view, top view, and the corresponding simulated electron diffraction pattern of the (11, 4)-R SWNT, respectively. The blue helix along a zigzag lattice direction is right-handed, which is also the zigzag helix that adopts the minimum screw pitch among the three zigzag helices. Therefore, the SWNT is right-handed. (d-f) Side view, top view, and the corresponding simulated diffraction pattern of the (4, 11)-L SWNT. The red helix, which has the smallest pitch among the three zigzag spirals, is left-handed, thus resulting in the assignment of the SWNT to be left-handed.

Figure S6 shows how to determine the handedness of a chiral SWNT in reciprocal space. Figure S6 a, b and Figure S6d, e are the side and top views of a (11,4)-R SWNT and a (4, 11)-L SWNT, respectively. As discussed in Figure S1, the handedness of a SWNT can be determined by the handedness of the spiral along a zigzag direction that has the smallest screw pitch. The spirals that have the smallest pitch are marked by the blue and red lines in (b) and (e), which are right-handed and left-handed, respectively. Figure S6c and f display the simulated diffraction patterns corresponding to the (11, 4)-R SWNT and the (4, 11)-L SWNT, respectively. Taking Figure S6c as an example, the upper wall of the blue spiral in (b) contributes to the pair of the reflection streaks marked by the blue dashed line. The lower wall

of the blue spiral leads to the other pair of the reflection streaks labeled by the orange dashed line. The orange line needs to be rotated counterclockwise with a twist angle of $<60°$ to turn into the blue line. In contrast, for the diffraction pattern of the (4, 11)-L SWNT, the upper and lower walls of the red spiral lead to the pairs of the reflection streaks that are marked by the blue and orange dashed lines in Figure S6f, respectively. In this case, the orange line needs to be rotated clockwise to transform into the blue line, which is just the opposite operation compared with the case for the right-handed SWNT.

To sum up, the determination method of the handedness of a chiral SWNT in reciprocal space involves the following three steps: (1) Find the two pairs of the reflection streaks in a FFT image or a diffraction pattern that are contributed from the spiral along a zigzag direction of a SWNT that adopt the smallest screw pitch. These streaks locate the farthest away from the equatorial layer line[3], which are commonly labelled as L_1, similar as the markers in Figure 2b; (2) Label the pair of the reflection streaks corresponding to the upper wall of the spiral and connect them using a blue dashed line. Conduct a similar operation for reflection streaks contributed by the lower wall of the spiral and connect the two streaks by an orange dashed line. In this step, if SWNTs are on top of Gr in TEM, the brighter reflection streaks should correspond to the lower surface of SWNTs, while the dimmer ones are contributed by the upper wall of SWNT. If SWNTs are on the lower surface of Gr in TEM, the brighter streaks should be assigned to the upper wall of SWNT, while the dimmer ones belong to the lower wall of SWNT. (3) Rotate the orange line an angle less than 60° clockwise or counterclockwise to coincide with the blue line. If the rotation is counterclockwise, the SWNT is right-handed. If the rotation is clockwise, the SWNT is left-handed.

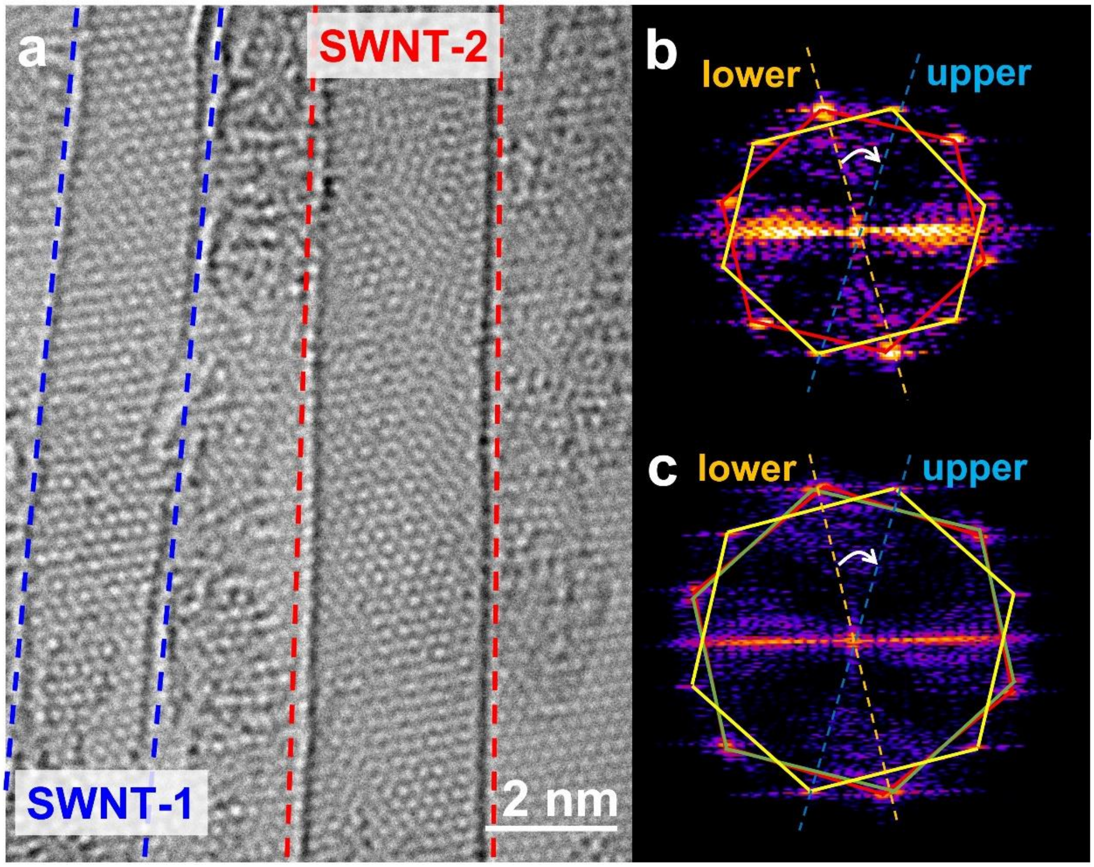

Figure S7. Determining the full structure of SWNTs locating closely to each other tube by tube. (a) AC-TEM image showing two SWNTs locating within 2 nm apart, labelled by blue and red dashed lines, respectively. (b) FFT image of SWNT-1. The yellow hexagon represents the reflexes from the SWNT upper wall, while the red hexagon represents the reflexes from both the SWNT lower wall and the underlying Gr. (c) FFT image of SWNT-2. The red hexagon represents the reflexes from Gr, which is quasi-superimposable with one set of SWNT reflections marked by the green hexagon.

The high spatial resolution of AC-TEM enables us to distinguish the full structuture of SWNTs tube by tube even when they situate only within several nanometers apart as long as they align with the underlying Gr. Figure S7a shows two SWNTs situating ∼2 nm apart. Figure S7b is the FFT image of SWNT-1, which shows that the lower wall of the SWNT and the underlying Gr is perfectly aligned, leaving only two groups of reflexes. Therefore, the complete structure of SWNT-1 can be determined based on the method introduced in Figure 2,

which is a (7, 19)-L SWNT. Figure S7c is the FFT image of SWNT-2. In this case, Gr reflexes are quasi-superimposable with a set of SWNT reflexes with an misorientation angle of only ～2° . If tolerating such a small angle deviation, the reflections from the SWNT upper and lower walls can be marked by the yellow and green hexagons, respectively. Therefore, the complete structure of SWNT-2 can be assigned as (10, 26)-L.

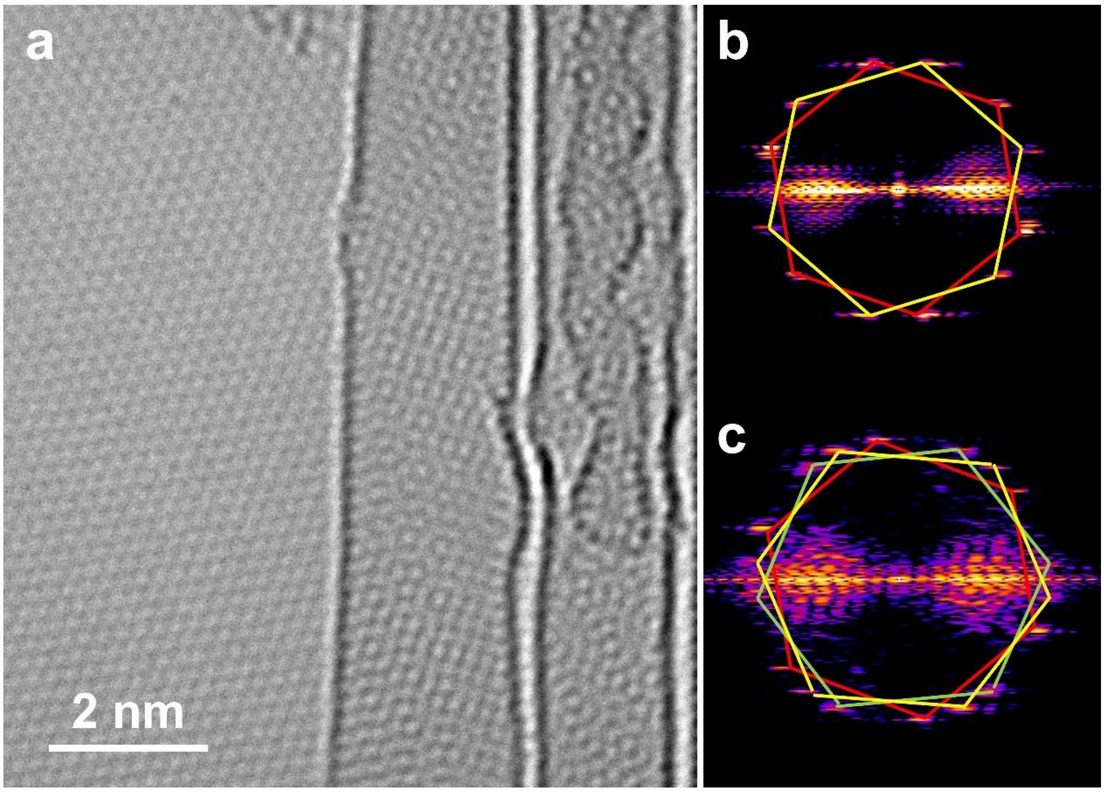

Figure S8. SWNT alignment in a bundle. (a) AC-TEM image of a bundle of two SWNTs. (b) FFT image of the left SWNT in (a). Two groups of reflexes are seen, representing good alignment between Gr and the SWNT lower wall. (c) FFT image of the right SWNT in (a). Three groups of reflexes are seen, indicating unparallel alignment between SWNT and the underlying Gr. The strong interaction between the two nanotubes hinders their independent orientation adjustment on Gr, thus resulting in the difficulty of the alignment of both two SWNTs with different chiral indices on Gr.

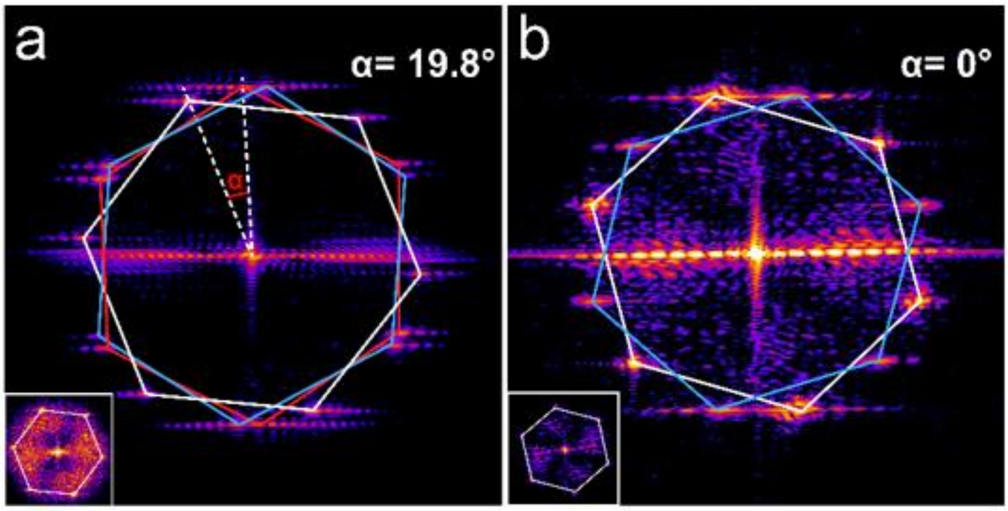

Figure S9. Determination of the stacking angle (α) in a SWNT/Gr heterostructure. (a, b) FFT images of two AC-TEM images having SWNT/Gr heterostructures, showing different stacking angles (α) of (a) 19.8°and (b) 0°. Insets show the FFT image of Gr surrounding the SWNT, which help confirm which set of reflexes correspond to Gr in the SWNT/Gr heterojunction region.

The stacking angle (α) between a SWNT and Gr in a heterostructure is determined in reciprocal space by conducting fast Fourier transformation of an AC-TEM image. As shown in Figure S9a, when three groups of reflexes are present, meaning that the SWNT does not align with Gr, α is defined as the smallest twist angle between the Gr reflexes (marked by the white hexagon) and one set of the SWNT reflexes (marked by red hexagon). In the case of panel a, α was measured to be 19.8°. When only two sets of reflexes are present at the heterostructure region, it means that the Gr reflexes coincide with one set of SWNT reflexes. indicating that the SWNT is perfectly aligned with Gr. In this case, α equals to 0°, as shown in Figure S9b.

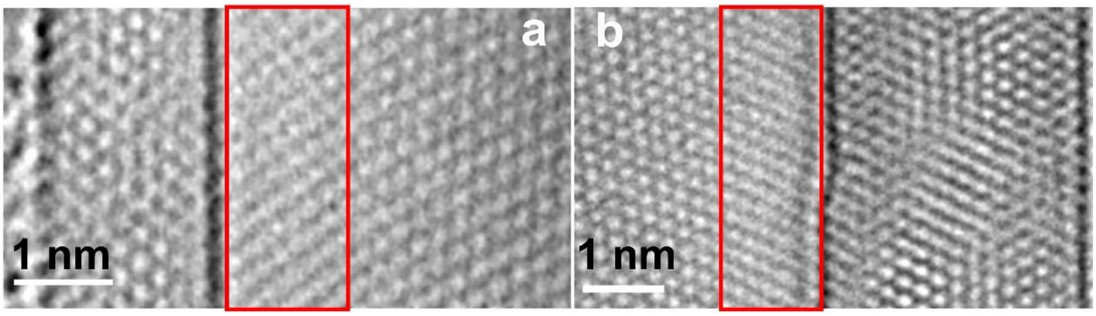

Figure S10. Out-of-plane deformation of Gr close to SWNTs. Two examples showing the blurry lattice of Gr when it is close to the SWNT, as highlighted by the red rectangles. The results indicate that the out-of-plane lattice deformation of Gr due to the loading of SWNT on its surface is a common phenomenon.

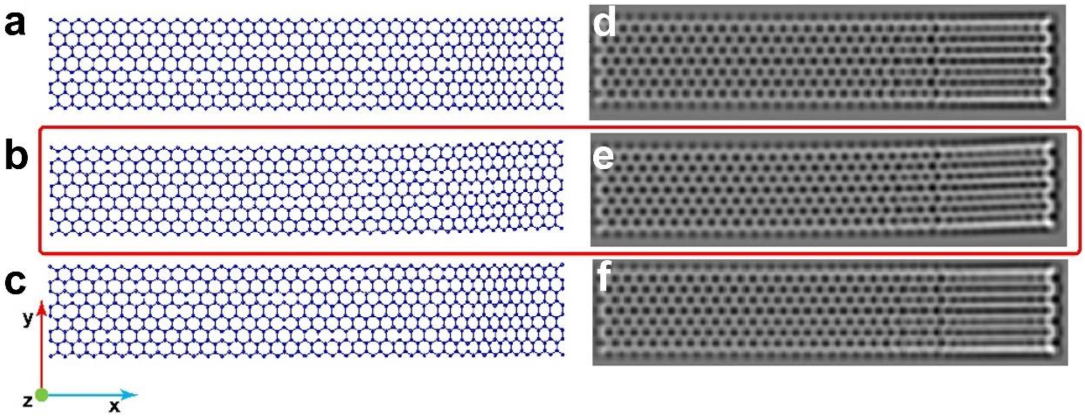

Figure S11. Simulated AC-TEM images corresponding to Gr with different types of out-of-plane distortion. The x-y plane is normal to the electron beam, while the z direction is parallel with the electron beam, which is also defined as the out-of-plane direction in this case. (a-c) Atomic models of Gr with different types of deformation. From top to bottom: the right end of the Gr ribbon is bent down by ~1.5 nm along the z direction without deformation along other directions. (a); the right end of the Gr ribbon is bent down along the z direction and the whole Gr ribbon is rotated 2° around the x axis (b); the right end of the Gr ribbon is bent down along the z direction, and the whole Gr ribbon is rotated 2° around the y axis (c). (d-f) Simulated AC-TEM images based on the atomic models in (a), (b), and (c), respectively.

Figure 3c and Figure S10 both display several examples of Gr lattice blur when it approaches the SWNT wall. The clear hexagonal lattice transfroms into lines with addition upward or downward bending. We construct several Gr atomic models with different types of deformation to try unclosing the reason of the such lattice blue phenomonon. The Gr nanoribbon atomic model in Figure S11a is slight curved downwards (along the z axis) at the right end. All the other regions is flat and undistorted. Such model mimics the out-of-plane deformation of Gr when it approaches a SWNT. In this case, the simulated AC-TEM image in Figure S11d shows the transformation from the clear hexagon lattice to only horizontal lattice lines from left to right, which is consistent with the main phenomena obseved in the experiments. However, the only out-of-plane distortion along z axis cannot induce the upward or doward bending of the horizontal lattice lines, which slightly deviates from the scenario observed in Figure 3c. This may indicate that Gr has deformation along other directions with repect to the electron beam besides the z direction. Therefore, Figure S11b and c construct atomic models having the whole Gr ribbon to be rotated 2° around the x axis and the y axis, respectively, together with the out-of-plane deformation along the z axis at the right end. Figure S11e is well consistent with the experimental observation in Figure 3c, indicating that the slight tilting of the whole Gr plane leads to the lattice line bending. This phenomenon is common is the TEM imaging, since the heterostructures may involve ripples due to the transfer process.

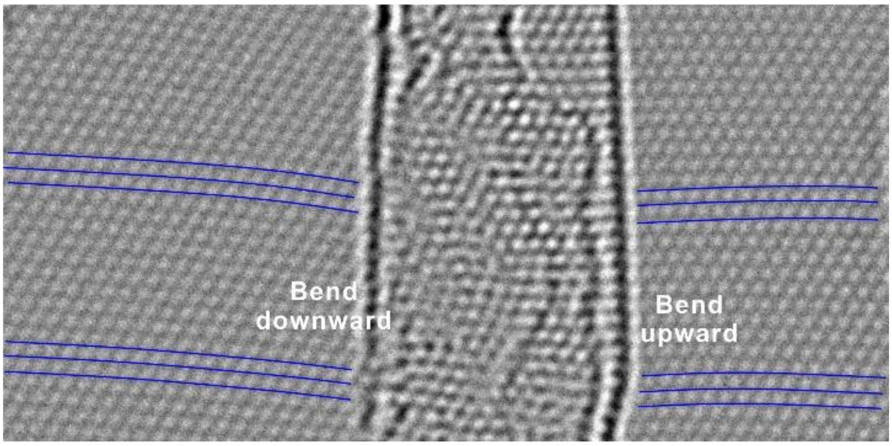

Figure S12. **AC-TEM image showing the Gr lattice bending on both sides of a SWNT**. It can be seen that the Gr lattice on the left side of the SWNT bends downward, while the Gr lattice on the right side of the SWNT shows a similar bending but in the opposite direction (symmetrical to the left side), as highlighted by blue lines. This phenomenon supports that the Gr lattice bending observed close to the SWNT stems from the SWNT induced Gr curvature instead of image drift.

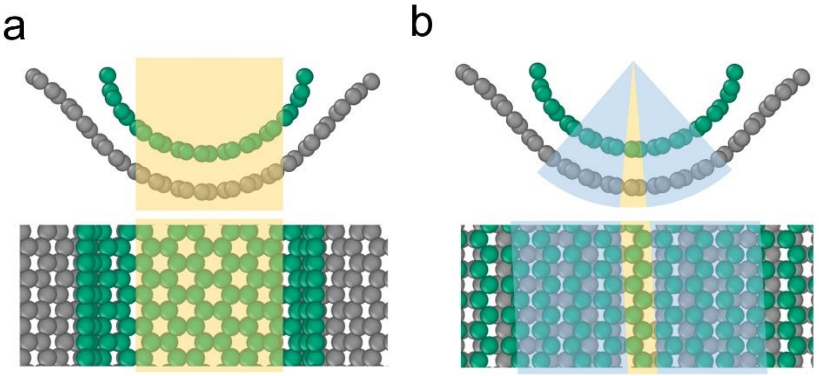

Figure S13. Interfacial stacking configurations of (12,12) SWNT/Gr van der Waals heterostructures. a) Parallel projection showing the interface configuration of a relaxed (12,12) SWNT/Gr heterostructure with a “surface contact” mode, in which a large area at the interface displays an AA stacking configuration (yellow-shaded region). b) Central projection of the (12,12) SWNT/Gr heterostructure interface, where most areas adopt staggered stacking (close to AB stacking, blue-shaded region) with only one column atoms having AA stack (yellow-shaded region).

We conducted additional DFT calculations to investigate the stacking configuration of a (12,12) SWNT (armchair tube) on Gr. As shown in Figure S13, the (12,12) SWNT/Gr heterostructure is AA stacking in the parallel projection view, while in the central projection view, the AA stacking only exists at the bottom of the nanotube with a width of one column of atoms. As the position is progressed to both sides of the nanotube, the interlayer stacking structure evolves into a near-AB stacking with staggered atoms. These results are similar to those obtained in the (13,11) SWNT/Gr heterostructure, indicating that the stacking configuration reported in the curved (13,11) SWNT/Gr interface is universal for other SWNTs on Gr.

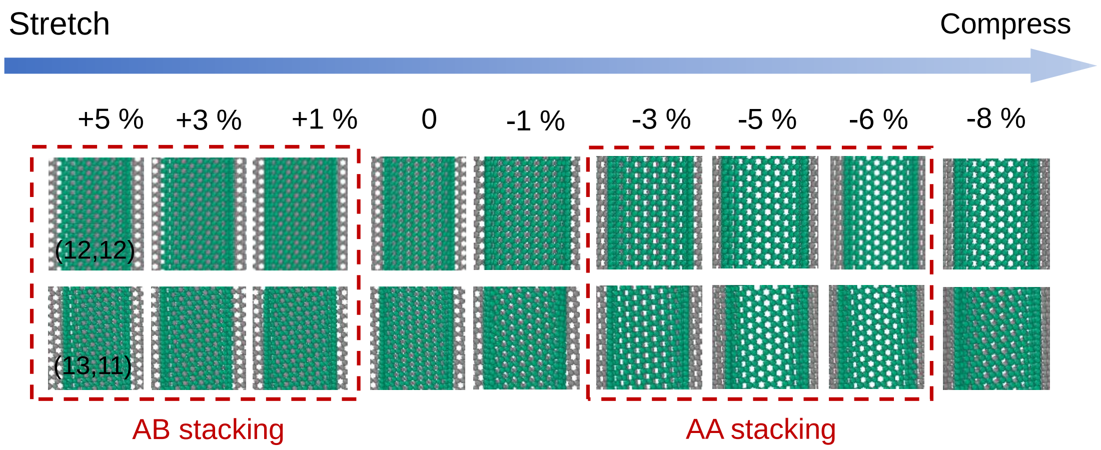

Figure S14. Interfacial stacking models of (12,12) SWNT/Gr heterostructure and (13,11)-R SWNT/Gr heterostructure under different strains of graphene.

We applied molecular dynamics simulation and found that different strain levels of Gr do impact the stacking structure at the SWNT/Gr interface. We took a (12,12) SWNT (an armchair nanotube) and a (13,11)-R SWNT (a chiral nanotube) as two examples. As shown in Figure S14, when strain in Gr gradually changes from stretching to compressing (the compression ratio changes from +5 % to -8 %), the interfacial configurations of both two different SWNT/Gr heterostructures evolve from the initial AB stacking to AA stacking in the projection view. It indicates that subtle lattice change of Gr could affect the energetically favorable structure at the SWNT/Gr heterostructure interface. Moreover, when the compressive strain of Gr is greater than 5 %, the heterostructure reaches a typical "surface contact" mode with a significant interfacial curvature, which has been summarized in Figure 3e.

Table S1. Electrical property classification of SWNTs based on their chiral indices.

	**SWNT arrays**	**Random SWNTs**	**Total**
**Semiconducting**	27	5	32
**Metallic**	13	9	22

It has been well documented that, for a given (n,m) SWNT, if n-m=3k (k=0,1,2,…), the nanotube is metallic. If n-m=3k±1 (k=0,1,2,…), the nanotube is semiconducting. Based on this rule, we resolved the chiral indices of 54 SWNTs and classifies their electrical properties into two groups, as shown in Table S1. It is worth noting that the determination of the chiral indices of SWNTs does not require the tube lower wall to be epitaxially aligned with Gr. Therefore, the sample size for the SWNT electrical property analysis here is bigger than that for the handedness analysis in Figure 4c. Among the 54 SWNTs, 40 tubes are from the SWNT array sample, while 14 tubes are from the random SWNT sample. For SWNTs from arrays, 27 tubes are semiconducting, and 13 tubes are metallic. The ratio between semiconducting and metallic SWNTs is very close to 2:1, which obeys the natural distribution of the SWNT electrical property. However, the semiconducting to metallic SWNT ratio of random SWNTs is 5:9, which deviates from the natural distribution. The potential reasons might stem from the small number of analyzed samples of this type and the CVD growth method that is distinct from the SWNT array, which may induce metallic nanotube enrichment.

References

[1] G. G. Samsonidze, A. Grüneis, R. Saito, A. Jorio, A. G. Souza Filho, G. Dresselhaus, M. S. Dresselhaus, *Phys. Rev. B* **2004**, *69*, 205402.

[2] K. Mustonen, A. Hussain, C. Hofer, M. R. A. Monazam, R. Mirzayev, K. Elibol, P. Laiho, C. Mangler, H. Jiang, T. Susi, E. I. Kauppinen, J. Kotakoski, J. C. Meyer, *ACS Nano* **2018**, *12*, 8512.

[3] L. C. Qin, *Phys. Chem. Chem. Phys.* **2007**, *9*, 31.
